# Bidirectional associations between psychosocial well-being and body mass index in European children: longitudinal findings from the IDEFICS study

**DOI:** 10.1186/s12889-016-3626-4

**Published:** 2016-09-08

**Authors:** Monica Hunsberger, Susanna Lehtinen-Jacks, Kirsten Mehlig, Wencke Gwozdz, Paola Russo, Nathalie Michels, Karin Bammann, Iris Pigeot, Juan Miguel Fernández-Alvira, Barbara Franziska Thumann, Dénes Molnar, Toomas Veidebaum, Charalambos Hadjigeorgiou, Lauren Lissner, on behalf of the IDEFICS Consortium

**Affiliations:** 1 Section for Epidemiology and Social Medicine (EPSO), The Sahlgrenska Academy, University of Gothenburg, Box 453, 40530 Gothenburg, Sweden; 2 School of Health Sciences (HES), FI-33014 University of Tampere, Tampere, Finland; 3 Department of Intercultural Communication and Management, Copenhagen Business School, POR/18.B-1.118, Copenhagen, Denmark; 4 Institute of Food Sciences, CNR Via Roma 64-83100, Avellino, Italy; 5 Ghent University, Ghent, De Pintelaan 185 4 K3, 9000 Belgium; 6 Institute for Public Health and Nursing Research, Faculty of Human and Health Sciences (FB 11), University of Bremen, Grazer Str. 2a, 28359 Bremen Germany; 7 Institute of Statistics, Faculty of Mathematics and Computer Science, University of Bremen, Bremen, Germany; 8 Centro Nacional de Investigaciones Cardiovasculares Carlos III Madrid, Spain and GENUD (Growth, Exercise, Nutrition and Development) Research group, University of Zaragoza, Madrid, Spain; 9 Leibniz Institute for Prevention Research and Epidemiology, BIPS, Achterstr. 30, 28359, Bremen, Germany; 10 Department of Pediatrics, Medical Faculty, University of Pécs, H-7623 Pécs, József A. u. 7, Hungary; 11 National Institute for Health and Development, Hiiu 42, 11619 Tallinn, Estonia; 12 Research and Education Institute of Child Health, Nicosia, Cyprus

**Keywords:** Childhood overweight, European cohort, Health-related quality of life, KINDL®, Strengths and Difficulties Questionnaire

## Abstract

**Background:**

The negative impact of childhood overweight on psychosocial well-being has been demonstrated in a number of studies. There is also evidence that psychosocial well-being may influence future overweight. We examined the bidirectional association between childhood overweight and psychosocial well-being in children from a large European cohort.

The dual aim was to investigate the chronology of associations between overweight and psychosocial health indicators and the extent to which these associations may be explained by parental education.

**Methods:**

Participants from the IDEFICS study were recruited from eight countries between September 2007 and June 2008 when the children were aged 2 to 9.9 years old. Children and families provided data on lifestyle, psychosocial well-being, and measured anthropometry at baseline and at follow-up 2 years later. This study includes children with weight, height, and psychosocial well-being measurements at both time points (*n* = 7,831). Psychosocial well-being was measured by the KINDL® and Strengths and Difficulties Questionnaire respectively. The first instrument measures health-related quality of life including emotional well-being, self-esteem, parent relations and social relations while the second measures well-being based on emotional symptoms, conduct problems and peer-related problems. Logistic regression was used for modeling longitudinal associations.

**Results:**

Children who were overweight at baseline had increased risk of poor health-related quality of life (odds ratio (OR) = 1.23; 95 % confidence interval (CI):1.03–1.48) measured 2 years later; this association was unidirectional. In contrast to health-related quality of life, poor well-being at baseline was associated with increased risk of overweight (OR = 1.39; 95 % CI:1.03–1.86) at 2 year follow-up; this association was also only observed in one direction. Adjustment for parental education did not change our findings.

**Conclusion:**

Our findings indicate that the association between overweight and psychosocial well-being may be bidirectional but varies by assessment measures. Future research should further investigate which aspects of psychosocial well-being are most likely to precede overweight and which are more likely to be consequences of overweight.

## Background

Childhood overweight is pervasively stigmatized in Western society, which may lead to psychological consequences [1, 2]. A number of studies have demonstrated that obesity and mental health disorders are comorbid conditions in adolescents [3–5] and adults [6–9]. There is also growing support for a bidirectional relationship [10–13]. However, in a systematic review of obesity and depression Luppino et al. reported that depression predicted the odds of future obesity but not overweight, mainly in adult populations [6]. Further, this same study reported that the cross-sectional association between depression and overweight was more pronounced in Americans than in Europeans [6].

While most previous prospective studies of depression and body weight have been conducted in adults, Herva et al. [14] studied European teens, and one study in the United States studied 11-year olds [15]. It is important to keep in mind that a child may experience poor well-being long before a clinical diagnosis of depression [16]. Childhood weight status and psychosocial well-being are associated with each other cross-sectionally [17, 18]; however, as in adults, less is known about the longitudinal association [19]. A reasonable foundation exists to hypothesize that the relationship between obesity and psychological well-being could be bidirectional, that is, obesity increasing the risk of future poor psychosocial well-being and even mental health problems and poor childhood psychosocial well-being increasing the risk of future obesity [6, 19]. Mental distress and/or behavioral problems [20–22] have been identified as risk factors for future obesity. Also, childhood obesity has been observed to be associated with future mental health problems such as depression [23] and emotional problems [24]. However, other studies have found no longitudinal associations between emotional problems and childhood obesity [25, 26].

Psychological health and health-related quality of life have been much less studied in relation to obesity than lifestyle factors [27, 28], although they may have fundamental importance in the etiology or/and consequences of obesity. Good health-related quality of life, defined as a state of psychological wellness that is free from depression and behavioral or emotional problems, [27] could be protective of future overweight or may be a consequence of current weight status. Similarly, psychological health or well-being, which describes individuals’positive emotions or satisfaction with life, may also be a cause or consequence of overweight [29]. Two instruments that can be used to measure psychosocial health include the KINDL® and the Strengths and Difficulties Questionnaire. These two instruments capture different aspects of well-being and are only partially redundant. It is potentially important for prevention as well as treatment of obesity to better understand temporal relationships with different indicators of psychosocial health.

Socioeconomic conditions are well-known determinants of both obesity and psychosocial health in early life [30]. Low-income children are also more prone to exhibit psychological and behavioral problems as a result of exposure to psychological stressors [28]. Thus, the dual aim of this study is to investigate the chronology of associations between overweight and psychosocial health indicators, and the extent to which these associations may be explained by parental education, a proxy for socioeconomic position.

## Methods

Participants from the Identification and prevention of Dietary and lifestyle induced health Effects In Children and infants Study (IDEFICS) is a prospective cohort study with an embedded intervention, including children from eight centers in Europe (Belgium, Cyprus, Estonia, Germany, Hungary, Italy, Spain and Sweden). The aim of the IDEFICS study was to assess the health status of European children with special focus on overweight and related co-morbidities. From September 2007 to June 2008, 16,228 children aged 2 to <10 years old underwent baseline examinations, followed by a setting-based community-oriented intervention with key health messages about diet, physical activity, sleep, and spending time together as a family. From September 2009 to March 2010, the majority of these children participated in a 2-year follow-up examination.

### Data collection

The details of the study design and instruments can be found in Ahrens et al. [31]. In brief, during baseline, children were recruited via schools and kindergartens. According to a standardized study protocol, parents reported socio-demographic, behavioral, medical, nutritional, family life and other lifestyle information, which was complemented by physical examinations of the children including anthropometry, blood pressure, and a number of other biological measures. As attrition is common in longitudinal studies, a detailed analysis of participants lost between baseline and 2-year follow-up was conducted [32]. Children attending the baseline examination who had a migrant background, lower parental education, poorer well-being, and overweight were more likely to not participate in follow-up examinations [32]. Descriptive statistics have been published for the entire cohort, including psychosocial health and anthropometry (for further details refer to [33]). The present study includes 7,831 children with anthropometric and psychosocial well-being measurements at both time points.

#### Psychosocial health measures

Psychosocial health was assessed by two instruments, both at baseline and 2-year follow-up. Health-related quality of life was assessed with the KINDL® questionnaire and well-being was assessed with the Strengths and Difficulties Questionnaire.

### Health-related quality of life

KINDL® was originally designed for measuring health-related quality of life in children and adolescents with disabilities and later validated for healthy populations [34]. The instrument was tested in 13 European countries for cross-cultural validity [35]. The IDEFICS study included a version that was developed for parental response on behalf of children and adolescents between 7 and 17 years of age. Parents/guardians reported on their children’s emotional well-being, self-esteem, parent relations and their social contacts. Each of these dimensions consists of four items. Using self-esteem as one example, the statements include: During the last week my child… (1) *had fun and laughed a lot*, (2) *didn’t feel much like doing anything,* (3) *felt alone,* and (4) *felt scared or unsure of itself* [34]. The answer categories ranged from 0 ‘not at all’ to 3 ‘often or always’. For calculating health-related quality of life, items were reversed where necessary, e.g., items (2), (3) and (4) in the self-esteem dimension; we then calculated the sum-scores for each dimension and a total composite score with potential values ranging from 0 to 48 – such that high values indicate a better health-related quality of life. For investigating the association with overweight including obesity, we dichotomized the composite score which ranged from 15–48 in our cohort by subdividing the composite score into four quintiles and assigning only those in the lowest quintile to poor health-related quality of life.

### Well-being

The well-being indicator used here is based on the Goodman et al. Strengths and Difficulties Questionnaire, which has been widely used in epidemiological studies to assess emotional and behavioral difficulties in children aged 4 to 16 years [36]. The IDEFICS study used the informant-rated version in which parents filled in the questionnaire on the child’s behalf. The Strengths and Difficulties Questionnaire score includes three dimensions: emotional symptoms, conduct problems and peer problems with 5, 3 and 5 items respectively from the 5 dimension scale. Parents responded to a series of statements with answer categories from 0 ‘not true’ to 2 ‘certainly true’. The total score ranges from 0 to 30 where a high value indicates more difficulties or life struggles. In accordance with Goodman et al. the composite score was divided into three categories: 0 – 11.25 ‘inconspicuous’, >11.25 – 14.25 ‘borderline’ and >14.25 ‘abnormal’. In this analysis we created a dichotomized variable consisting of inconspicuous, or no detectable poor well-being, versus poor well-being, which includes both borderline and abnormal categories.

### Anthropometric measurements

Weight was measured using an electronic scale (BC 420 SMA; Tanita Europe GmbH) to the nearest 0.1 kg in light underclothing. Height was measured to the nearest 0.1 cm using a stadiometer (Seca 225). BMI was calculated and converted to BMI-z-scores according to IOTF 2012 criteria [37]. In this study, children were categorized as either non-overweight (including normal weight and underweight) or overweight (referring to overweight including obesity).

### Covariates

Date of birth reported by parents was used to calculate the child’s exact age at baseline examination. Children were further classified as pre-school age (2 years old to <6 years old) or school age (6 years old to <10 years old). Socioeconomic position of the family was determined by a proxy measure using the highest educational level achieved in the household, classified according to the International Standard Classification of Education (ISCED) [36]. The original levels were defined as low (levels 1 and 2; ≤9 years of education), low-medium (level 3), medium (level 4) and high (levels 5 and 6). We further dichotomized those with high education (levels 5 and 6; ≥2 years of education after high school) and those without (levels 1–4) for analyses, hereafter referred to as lower and higher ISCED.

### Statistical analysis

Descriptive statistics are given for the prevalence of all three indicators of poor health: overweight, poor health-related quality of life and poor well-being at baseline and at follow-up. Background characteristics including age, sex, parental education, and country by each health indicator are shown by prevalence and row percentages and compared using chi-square tests.

Associations between weight status and both dichotomized indicators of poor psychosocial health were analyzed using chi-square tests, and mixed effects logistic regressions adjusted for age, sex, and ISCED as fixed effects, and survey country as random effects. In separate models we tested for potential effect modification by country, age, and child’s sex, as well as interactions between poor health-related quality of life and poor well-being, by including product terms in the models. To further examine how poor psychosocial health at baseline predicted incident overweight at follow-up we performed mixed effects logistic regression of overweight status at follow-up as a function of poor psychosocial health (poor health-related quality of life or poor well-being) at baseline in the subsample of children without overweight at baseline (Table 3a, model 1a and 1b). Conversely, we calculated whether overweight at baseline predicted incident poor psychosocial health in children without poor psychosocial health at baseline (Table 3a, model 2 and 3). We also investigated whether absence of poor psychosocial health predicted recovery from overweight in the subsample of overweight children at baseline (Table 3b, model 1a and 1b), or whether no overweight at baseline predicted recovery from poor psychosocial health in the subsample of children with poor psychosocial health at baseline (Table 3b, model 2 and 3). In our analyses, we also adjusted for the variable describing whether each child was in the intervention or control community.

Odd ratios (OR) with 95 % confidence intervals (CI) are reported for logistic regression analyses. All statistical tests were two-sided, and results with *p*-values < 0.05 were considered as statistically significant without adjustment for multiple comparisons.

Statistical analyses were conducted with STATA IC version 11.2 (StataCorp College Station, Texas 77845 USA).

## Results

Participant characteristics including presence of overweight, poor health-related quality of life and poor well-being at baseline and 2-year follow-up are shown in Table 1. Girls and boys were similarly represented in the sample. At baseline, 1419 out of 7831 children (18 %) were overweight. School children were more likely than pre-schoolers to be overweight. Prevalence of overweight at baseline varied by survey country; overweight was lowest among children in Belgium (6.1) and the highest among children in Italy (42.5 %). The prevalence of overweight as well as poor psychosocial health was lower in children whose parents had higher levels of education (*p* < 0.009 for all three associations) at baseline. There was no difference between intervention and control regions with regards to prevalence of overweight at follow-up.

**Table 1:** Descriptive characteristics in analytic sample (*n* = 7,831 measured at baseline, 2007/08 and 2 year follow-up, 2009/10), in subjects with poor health outcome (overweight including obesity (OWOB), poor well-being (PWB), and poor health related quality of life (PHRQL)

	Poor health outcome at two time points					
	OWOB, n (row %)		PWB, n (row %)		PHRQOL, n (row %)	
Year	2007/2008 (*n* = 1,419)	2009/2010 (*n* = 1,790)	2007/2008 (*n* = 616)	2009/2010 (*n* = 586)	2007/2008 (*n* = 2,380)	2009/2010 (*n* = 2,436)
Background variable						
Sex						
Boys	661 (16.7)	873 (22.0)	370 (9.3)	342 (8.6)	1,259 (31.8)	1,291 (32.6)
Girls	758 (19.6)	917 (23.7)	246 (6.4)	244 (6.3)	1,121 (29.0)	1,145 (29.6)
Age						
Pre-school 2- < 6 years	430 (12.3)	609 (17.4)	262 (7.5)	218 (6.2)	874 (25.0)	879 (25.1)
School age 6- < 10 years	989 (22.8)	1,181 (27.2)	354 (8.2)	368 (8.5)	1,506 (34.7)	1,557 (35.9)
Parental education (ISCED)						
Level 1	167 (37.8)	183 (44.7)	62 (15.2)	60 (14.7)	173 (39.1)	148 (36.2)
Level 2	569 (21.4)	701 (27.3)	228 (8.9)	207 (8.1)	883 (33.2)	838 (32.6)
Level 3	208 (15.5)	283 (20.6)	110 (8.0)	110 (8.0)	373 (27.8)	438 (31.8)
Level 4	460 (13.9)	594 (17.6)	197 (5.8)	198 (5.9)	918 (27.6)	975 (28.8)
Country						
Belgium	59 (6.1)	95 (9.8)	79 (8.2)	100 (10.3)	205 (21.2)	236 (24.4)
Cyprus	197 (25.1)	250 (31.9)	84 (10.7)	71 (9.0)	364 (46.4)	333 (42.4)
Estonia	158 (14.3)	186 (16.9)	79 (7.2)	89 (8.1)	270 (24.5)	356 (32.3)
Germany	103 (13.2)	125 (16.1)	73 (9.4)	72 (9.3)	191 (24.6)	166 (21.3)
Hungary	115 (13.0)	174 (19.7)	69 (7.8)	59 (6.7)	457 (51.8)	373 (42.2)
Italy	471 (42.5)	567 (51.1)	112 (10.1)	90 (8.1)	431 (38.9)	408 (36.8)
Spain	192 (19.3)	249 (25.0)	94 (9.5)	75 (7.5)	259 (26.0)	360 (36.2)
Sweden	124 (10.3)	144 (11.9)	26 (2.5)	30 (2.5)	203 (16.8)	204 (16.9)

Footnote: Overweight including obesity (OWOB), defined according to Cole 2012 and poor health related quality of life (PHRQOL) measured by KINDL® and poor well-being (PWB) measured by Strengths and Difficulties Questionnaire. Statistical testing included *χ* ^2^-test of differences in the prevalence of a poor health outcome (OWOB, PHRQOL, PWB, at baseline and 2 year follow-up) between categories of each of the 4 background variables (sex, pre-school v. school age, parental education and country). All other associations were statistically significant, except for: sex and prevalent OWOB 2009/2010, *p* = 0.07 and pre-school versus school age and PWB 2007/2008, *p* = 0.27

In Table 2 we show cross-sectional and prospective associations between overweight and both dichotomized indicators of poor psychosocial health (poor health-related quality of life and poor well-being) in the whole sample. Regarding poor health-related quality of life, none of the cross-sectional or longitudinal associations with overweight was statistically significant. In contrast, poor well-being at baseline predicted overweight at follow-up (OR = 1.22; 95 % CI:1.00–1.48), and poor well-being and overweight were cross-sectionally associated at follow-up (OR = 1.26; 95 % CI:1.03–1.54). Overweight at baseline was not statistically significantly associated with poor well-being at baseline, nor at follow-up.

**Table 2:** Cross-sectional and longitudinal associations between overweight including obesity (OWOB, and two dichotomized indicators of psychosocial health, poor health related quality of life (PHRQOL), and poor well-being (PWB), in the entire sample of children (*n* = 7,831) with measures at baseline (2007/08) and at follow-up (2009/10)

	PHRQOL		PWB	
Study year	2007/08	2009/10	2007/08	2009/10
	OR (95 % CI)^a^	OR (95 % CI)^a^	OR (95 % CI)^a^	OR (95 % CI)^a^
OWOB at baseline (2007/08)	1.03 (0.90, 1.17)	1.10 (0.96, 1.25)	1.18 (0.95, 1.46)	1.04 (0.83, 1.30)
OWOB at follow-up (2009/10)	1.00 (0.89, 1.14)	1.07 (0.95, 1.21)	1.22 (1.00, 1.48)	1.26 (1.03, 1.54)

Footnotes: OR (95 % CI): odds ratio (95 % confidence interval)

Overweight including obesity (OWOB), defined according to Cole 2012 and poor health related quality of life (PHRQOL) measured by KINDL® and poor well-being (PWB) measured by Strengths and Difficulties Questionnaire

^a^logistic regression of weight status on indicators of psychosocial health, adjusted for age, sex, parental education, intervention and country

From baseline to follow-up, a relatively small proportion of children changed from good to poor health status, with 570 (8.9) moving into the overweight category, 735 (12.9) changing from good to poor well-being, and 347 (4.8 %) who initially had good health-related quality of life changing to poor health-related quality of life.

To estimate the direct predictive effect of a health indicator at baseline on another health indicator at follow-up, we calculated incidence odds ratios of a particular poor health outcome at follow-up in subsamples of children without that poor health outcome at baseline (Table 3a). In a second set of analyses, we calculated odds ratios of recovery from poor health in subsamples of children with poor health at baseline (Table 3b).

**Table 3:** Bidirectional associations between overweight including obesity (OWOB) and two dichotomized indicators of psychosocial health, poor health related quality of life (PHRQOL) and poor well-being (PWB)

A: Incidence of poor health				
Model^a^	Predictor at baseline	Subsample	Outcome OR (95 % CI)	*P*-value
		No OWOB at baseline (*n* = 6,412)	OWOB at follow-up	
1a	PHRQOL		0.95 (0.78, 1.15)	0.58
1b	PWB		1.39 (1.03, 1.86)	0.03
1c^b^	PWB without PHRQOL		1.02 (0.56, 1.86)	0.95
	PWB with PHRQOL		1.68 (1.16, 2.42)	0.006
	PWB × PHRQOL		1.63 (0.81, 3.29)	0.17
		No PHRQOL at baseline (*n* = 5,451)	PHRQOL at follow-up	
2	OWOB		1.23 (1.03, 1.48)	0.02
		No PWB at baseline (*n* =7,213)	PWB at follow-up	
3	OWOB		1.05 (0.79, 1.41)	0.74
B: Recovery from poor health				
		OWOB at baseline (*n* = 1,397)	No OWOB at follow-up	
1a	Good health related quality of life		0.87 (0.61, 1.24)	0.44
1b	Good well-being		1.38 (0.84, 2.27)	0.21
		PHRQOL at baseline (*n* = 2,343)	No PHRQOL at follow-up	
2	No OWOB		1.04 (0.84, 1.30)	0.70
		PWB at baseline (*n* =597)	No PWB at follow-up	
3	No OWOB		1.33 (0.87, 2.02)	0.19

The top part of the table refers to the incidence of poor health in the subsample of children without symptoms at baseline (A). The bottom part of the table considers the recovery from poor health in the subsample of children with corresponding symptoms at baseline (B)

Footnotes: OR (95 % CI): odds ratio (95 % confidence interval)

Overweight including obesity (OWOB), defined according to Cole 2012 and poor health related quality of life (PHRQOL) measured by KINDL® and poor well-being (PWB) measured by Strengths and Difficulties Questionnaire

^a^Logistic regression adjusted for age, sex, parental education, intervention, and country

^b^Model 1c investigates the interaction between PWB and PHRQOL at baseline with respect to OWOB at follow-up in children without overweight at baseline

**Fig. 1:**
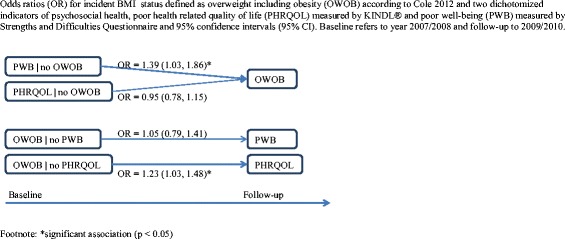
Odds ratios (OR) for incident BMI status defined as overweight including obesity (OWOB) according to Cole 2012 and two dichotomized indicators of psychosocial health, poor health related quality of life (PHRQOL) measured by KINDL® and poor well-being (PWB) measured by Strengths and Difficulties Questionnaire and 95 % confidence intervals (95 % CI). Baseline refers to year 2007/2008 and follow-up to 2009/2010

In Table 3 we present incidence of poor health (part A) and recovery from poor health (part B). In the adjusted models, poor health-related quality of life at baseline did not predict incidence of overweight at follow-up whereas overweight at baseline did predict poor health-related quality of life at follow-up, (OR = 1.23; 95 % CI:1.03–1.48). Poor well-being at baseline was predictive of overweight at follow-up (OR = 1.39; 95 % CI:1.03–1.86), whereas overweight at baseline did not predict poor well-being at follow-up (see Fig. 1). Examining the joint effects of poor health-related quality of life and poor well-being, we found that children with both poor health-related quality of life and poor well-being at baseline were most likely to become overweight at follow-up (OR = 1.68; 95 % CI:1.16–2.42), but the interaction between poor health-related quality of life and poor well-being was not statistically significant. Absence of poor health (no poor well-being, good health-related quality of life, or no overweight) did not predict recovery from poor health at follow-up in those with poor health at baseline. The results of the incidence analysis are summarized in Fig. 1.

## Discussion

This study found that children with poor well-being were more likely to become overweight 2 years later, whereas children with overweight at baseline were more likely to develop poor health-related quality of life. These associations could not be explained by education, a proxy for socioeconomic position. Further, among children with poor health-related quality of life, the association between poor well-being and overweight two years later was even stronger compared to those with good health-related quality of life, although the interaction between our two measures of psychosocial health predicting overweight was not statistically significant.

Our study is unique in that we were able to examine bidirectional associations between overweight and two measures of psychosocial health in a pan-European sample followed longitudinally. Although we were able to observe some longitudinal associations over the 2-year period, it will be of interest to re-examine these findings in the longer-term follow-up study that is ongoing. We employed the same measures at both time points and were therefore able to assess cross-sectional and longitudinal associations, including both prevalence and incidence of overweight and poor psychosocial health. However the study is not without limitations, including possible weaknesses in the proxy-reported indicators of psychosocial health used here. Further, the instruments were designed for children slightly older than some of the youngest children in our cohort and we examined three of the five dimensions in the original Strengths and Difficulties Questionnaire which has not been validated. Also, our measurements are dichotomized for bidirectional analyses and it is possible that stronger associations may have been detected using continuous variables. Finally, it is acknowledged that self-selection may have occurred in the initial 2 years of the study, such that participants with overweight and/or low psychosocial health may have been less likely to attend both examinations and if they did participate at baseline, might have excess risk of being lost to follow-up.

Our findings can be compared and contrasted with a study of the chronology of overweight and health-related quality of life conducted with 3,898 Australian children participating in the Longitudinal Study of Australian Children (LSAC), assessed at four time points between ages 4 and 11 years [38]. In contrast to our study, the LSAC assessed health-related quality of life using the pediatric quality of life inventory (PedsQL) rather than the KINDL®. Further, the LSAC examined a longer-term cumulative burden of poor health-related quality of life and overweight based on multiple examinations. However, our results can be compared in some regards. Findings from LSAC indicated that poor psychosocial health was associated with an increased risk of overweight by age 6–7 years with an odds ratio of 1.32. Similarly, we found an association between poor well-being and overweight over a 2-year period in our cohort aged 2–9 years at baseline with a similar odds ratio of 1.39. Though our measurement instruments differ, the LSAC study reported a strong relationship between poor social functioning and overweight. This dimension of the PedsQL includes five items (1. getting along with other children, 2. other kids not wanting to be his or her friend, 3. getting teased by other children, 4. not able to do things that other children his or her age can do, and 5. keeping up when playing with other children) which is most comparable to our measure of well-being based on the Strengths and Difficulties Questionnaire [34]. This is in support of our finding that poor well-being was associated with overweight 2-years later, although our time horizon and measures of well-being differ. The LSAC group further reported that overweight predicted poor psychosocial health most strongly for subscales reflecting social and emotional functioning [39]. This is similar to our finding that overweight at baseline was associated with poor health-related quality of life at 2-year follow up. The KINDL® instrument includes both emotional and friend relationship dimensions which may be comparable to the measures employed in the LSAC study for emotional and social well-being. In a subsequent study including 3197 children from the LSAC group of children 4–11 years of age, the Strengths and Difficulties Questionnaire was also used [40]. Here, the LSAC group reported that more cases of overweight predicted higher total difficulty scores using the same Strengths and Difficulties Questionnaire as used in our study. However, the LSAC group finding is in contrast with our finding that poor well-being at baseline was predictive of overweight at follow-up but overweight at baseline did not predict poor well-being at follow-up. The difference in our findings could be due to the fact that we considered only three of the five dimensions included in the Strengths and Difficulties Questionnaire.

Another study of changes in BMI and health related quality of life from childhood to adolescence collected data in children at three points in time: 1997 to 2000 and 2005 as part of the Health of Young Victorians longitudinal cohort study [41]. This study also used the PedsQL and calculated BMI z-scores from measured height and weight [41]. Williams et al. reported cross-sectional, inverse associations between lower health-related quality of life and higher BMI categories but did not find convincing evidence for causality in either direction [41]. However, the parent-reported total PedsQL score predicted high subsequent BMI, which is similar to our finding that poor well-being was associated with overweight 2 years later.

In a study of young people followed for 10 years in South Australia, health-related quality of life was lower among those in overweight and obese compared to normal weight participants. This study used two questionnaires; the Short-Form Health Survey (SF-36) and another quality of life instrument [11]. Although this study recruited older participants and used different indicators of well-being from our study, their finding that poor well-being in teens and young adults was predictive of overweight is in line our findings in younger children. Further, this study examined major depression and reported that those with an unhealthy BMI (underweight, overweight and obese) compared to a normal BMI suffered more often from major depression [11], which may indicate that those with comorbid overweight and poor psychosocial health early in life are at increased risk for major depression in the future. Although we were unable to measure this in IDEFICS our observed associations between overweight and psychosocial health mightbecome stronger as our cohort approaches adulthood.

In a 2008 review of 24 studies examining the relationship between obesity and depression, the authors concluded that there was a weak level of evidence supporting the hypothesis that overweight increases the incidence of depression [10]. Though we have studied children rather than adults and psychosocial well-being rather than clinical depression, our findings add to the sparse literature that attempts to examine causality between overweight and measures of psychosocial health. Again not considering directionality as we have, Blane combined data from over 33,000 individuals and concluded that depressed compared with non-depressed people are significantly more likely to be obese at follow-up [13]. However, in contrast to our cohort, most were adults and the youngest participants (9 years old) were the same age as our oldest participants. Our findings add to what little is known by investigating directionality within a younger cohort. A number of reviews have reported an association between overweight and various measures of psychosocial/mental health, including major depression, in children [3–6, 27, 28] but few have examined the bidirectional chronology of the relationship between overweight and psychosocial health in children.

Our finding that poor well-being increased the risk for overweight and that overweight increased the risk for poor health-related quality of life may reflect the complex relationship between psychosocial health and overweight. Parents of children who experienced early overweight were more likely to report poor health-related quality of life in their children 2 years later. Parental reports of poor well-being significantly predicted overweight 2 years later. Thus, it may be that children with these problems are more likely to develop overweight and then once a child is overweight he/she is more likely to have poor health-related quality of life. Further, since we found that in those children with poor scores for both indicators of well-being at baseline were the most likely to become overweight at the second measurement, it seems particularly important to identify children with poor psychosocial health early in life. Research with older youth and adults indicates that the comorbidities of poor psychosocial health and overweight place a person at increased risk for major depression. This suggests a strong need for multi-disciplinary interventions that are aimed not just at obesity prevention but also aimed at bolstering psychosocial health. Further, it may be equally important to utilize early screening for psychosocial health and overweight as a complement to what is often conducted in school-based settings; for example, dental, hearing and vision exams.

The present results may have important implications for future public health efforts. For example, identifying young children who may be suffering from poor well-being could allow for interventions aimed at primary prevention of overweight and secondary prevention of poor psychosocial health. Russell-Mayhew proposed a detailed model of the potential mechanisms acting between childhood overweight and quality of life, which may be useful in planning future interventions and research [27]. While we have investigated just two of the factors proposed in this model, emotional well-being and self-esteem as components of the KINDL® questionnaire, our findings support the existence of a bidirectional relationship between overweight and psychosocial well-being. Future research should seek to understand critical developmental periods in childhood as well as optimal methods for preventing both conditions of overweight and poor psychosocial well-being at an early age. Our research provides evidence that it is important to begin at a young age.

## Conclusion

In a large pan-European sample we have shown evidence for a complex bidirectional relationship between overweight and psychosocial well-being that is dependent on the instrument used. Poor well-being measured by the Strengths and Difficulties Questionnaire was associated with an increased risk for overweight 2 years later, whereas children who were overweight at the start were more likely to have developed poor health-related quality of life measured by KINDL® at 2-year follow-up. Studies with comparable measures of psychosocial health and designs that allow for bidirectional analyses are needed in order to better understand the mechanisms for relationships between overweight and comorbidities such as poor psychosocial health.
